# The paracondylar process: anatomical relationships and clinical implications

**DOI:** 10.1007/s00276-026-04009-3

**Published:** 2026-10-30

**Authors:** Beitris Chiu, Nigel Williams, Hong Kuan Kok, Hamed Asadi, Christen D Barras

**Affiliations:** 1 Faculty of Health and Medical Sciences, Adelaide University, Adelaide, South Australia Australia; 2 Royal Adelaide Hospital, Adelaide, South Australia Australia; 3 Paperscape, Adelaide, South Australia Australia; 4 Interventional Radiology Service, Northern Imaging Victoria, Northern Health, Victoria, Melbourne Australia; 5 NECTAR Research Group, Northern Health, Victoria, Melbourne Australia; 6 Faculty of Medicine, Dentistry and Health Sciences, University of Melbourne, Victoria, Melbourne Australia; 7 School of Health and Biomedical Sciences, RMIT University, Victoria, Melbourne Australia; 8 School of Medicine, Faculty of Health, Deakin University, Pigdons Road, Waurn Ponds, Victoria Australia; 9 Interventional Neuroradiology Service, Radiology Department, Austin Hospital, Victoria, Melbourne Australia; 10 Department of Radiology, Royal Adelaide Hospital, Adelaide, South Australia Australia

**Keywords:** Paracondylar process, Vertebral artery, Occipital vertebrae, Anatomic variant

## Abstract

**Purpose:**

The paracondylar process (PCP) is a rare and infrequently reported anatomical variant, where the incomplete separation of the lowest occipital sclerotome (proatlas) and uppermost cervical sclerotome results in a complete bony projection that meets with an epitransverse process of the atlas. We report a case that highlights the imaging and anatomical relationships of the PCP, particularly with the vertebral artery (VA), which has been given little attention in literature despite its important symptomatic and neurosurgical implications.

**Case presentation:**

A 55-year-old male presented with longstanding limitation to neck movement, left‑sided headaches and occasional dizzy spells. Cervical computed tomography (CT) imaging demonstrated a bony strut projecting inferiorly from the skull base, laterally adjacent to the left occipital condyle, articulating with the epitransverse process of C1. Though CT angiography was not performed, close examination of the course of the left vertebral artery on the soft tissue CT windows demonstrated an intimate relationship of the artery coursing immediately medial to the bony process in its V3 segment. We present a three-dimensional reconstruction prepared via segmentation and close reading of the soft tissue CT to illuminate the expected course of the vertebral artery and its’ relationship to the PCP.

**Conclusion:**

This case demonstrates the anatomical and clinical significance of the paracondylar process (PCP), with a particular focus on its relationship with the vertebral artery. Whilst rare, this finding carries important clinical implications if surgical resection is to be contemplated.

**Supplementary Information:**

The online version contains supplementary material available at https://doi.org/10.1007/s00276-026-04009-3.

## Introduction

The paracondylar process (PCP) is defined as an inferior extension of the jugular process of the occipital bone, typically articulating with the superior surface of a transverse process of the atlas [7]. A complete PCP is rare and has been infrequently reported in anthropological, radiological and cadaveric studies. This variant was present in just one of over 1300 skulls examined in a medieval graveyard in Canterbury (0.08%) [1]. In a study based on examination of plain film X-Rays of 4000 patients, 5 cases of PCP’s were reported (0.125%) [5]. Further, in an anatomical study of 692 specimens, 2 cases of PCP were reported (0.29%) [10].

The origin of the PCP is a matter for debate [9]. In the embryological theory, it is hypothesized that the PCP develops as the result of maldevelopment of the first cervical sclerotome around the 4th week of development in utero [7]. The basiocciput, which is one of three precursors of the occipital bone, develops into four occipital somites. The caudal portion of the 4th occipital somite fuses with the cranial portion of the 1st cervical somite to form the proatlas. The caudal half of the 1st cervical somite along with the cranial part of the second cervical somite form the atlas and odontoid process of the axis [7]. The PCP is thought to represent vestiges of the cranial half of the first cervical sclerotome, as a result of a process referred to as a caudal shifting, where a vertebrae takes on the characteristics of its caudal neighbour [7]. The resulting bony projection may be complete, meeting with an epitransverse process of the atlas (PCP); an isolated intervening bony segment (massa paracondylica); or, more frequently, a small skull base exostosis (tuberculum parcondylica) [8].

The alternative, less commonly reported hypothesis suggests that the PCP is a pathologic, acquired process due to excessive pull by the rectus capitis lateralis muscle, lateral atlanto-occipital ligament or trauma [9].

We report the case of a PCP, and discuss its important anatomical relationships with the aid of a three dimensional reconstruction prepared via segmentation and close reading of the soft tissue computed tomography (CT).

## Clinical presentation

A 55-year-old male right-handed male office worker presented with longstanding limitation to neck movement, left sided headaches and occasional dizzy spells for investigation. There was no history of trauma or neck manipulation.

The patient was referred for neurosurgical outpatient review. On examination, there was restriction of lateral flexion and leftward rotation. Neurological examination including positional tests for vertebrobasilar insufficiency was unremarkable.

**Fig. 1:**
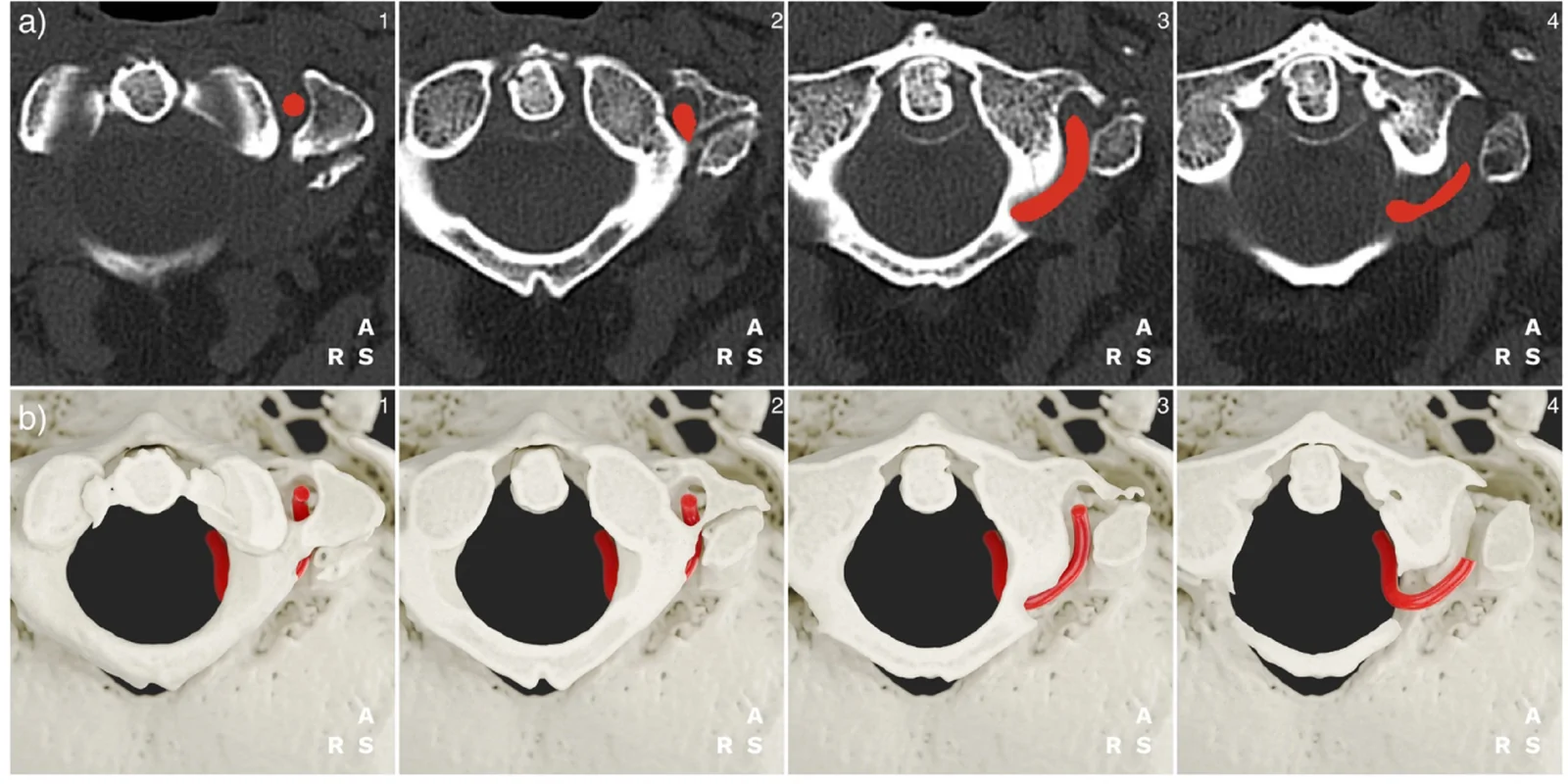
Axial slices** a** of the original CT and **b** the corresponding slices of the three-dimensional reconstructed model, demonstrating the estimated course of the VA, shown in red, in relation to the PCP. Images are caudocranial from left to right

**Fig. 2:**
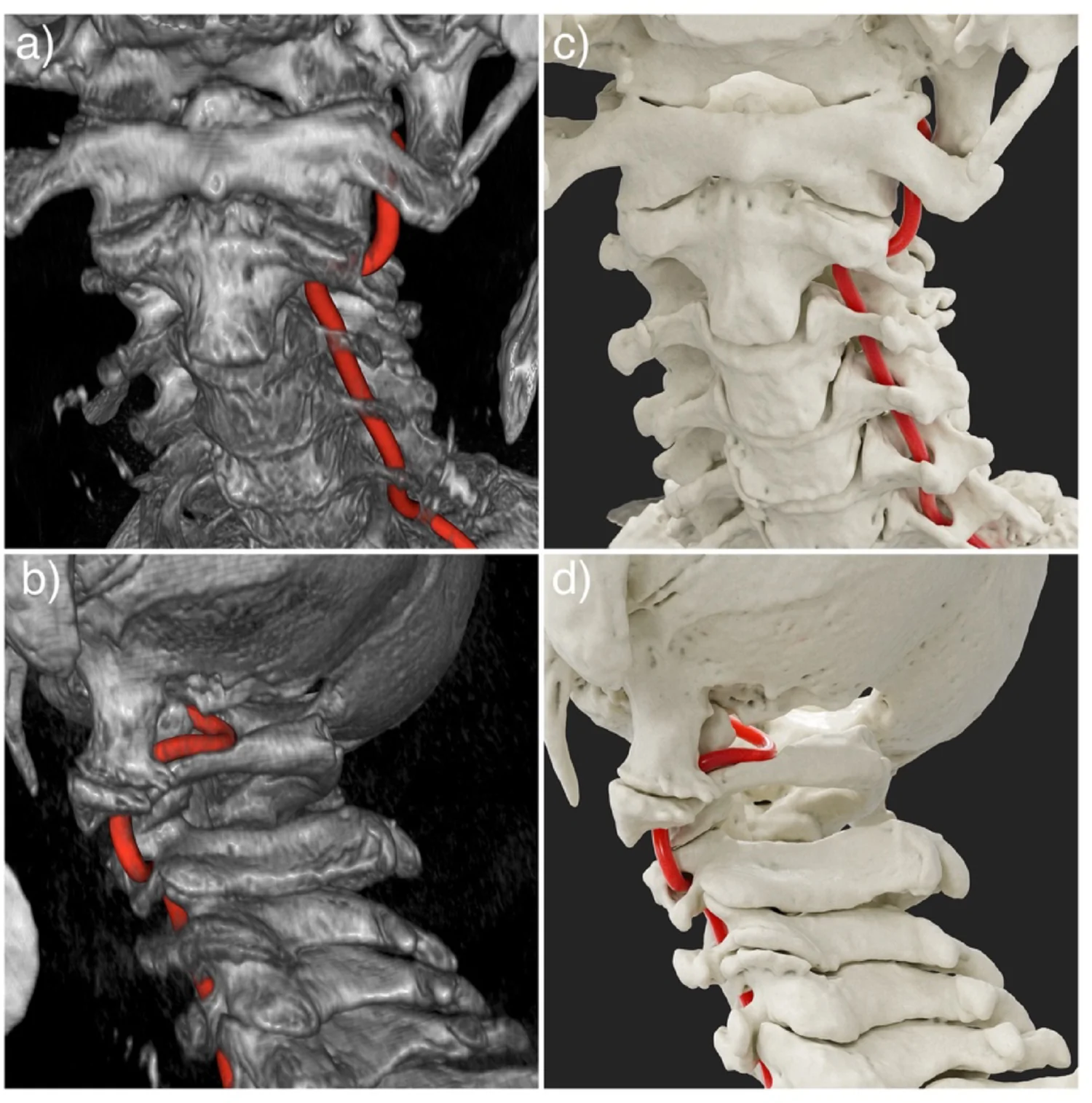
Shows the articulated PCP through** a** frontal and** b** left lateral views of the three-dimensional rendering of the original CT with corresponding** c** frontal and** d** left lateral views of the reconstructed model. The estimated course of the VA has been demonstrated in red

CT of the cervical spine demonstrated a bony strut projecting inferiorly from the skull base, arising laterally adjacent to the left occipital condyle (Fig. 2). The bony extension formed an articulation with an epitransverse process of C1. Though CT angiography was not performed as conservative rather than surgical management was ultimately deemed appropriate, close examination of the course of the left vertebral artery (VA) on the soft tissue CT windows demonstrated an intimate relationship of the artery coursing immediately medial to the bony process in its V3 segment (Fig. 1). The diagnosis of a left paracondylar process was made.

Following specialist discussion with the patient, non-surgical management was deemed appropriate.

## Discussion

A well-developed PCP, as presented in our case, is rare, with few reports published in medical literature [1].

As in this case, a PCP may result in osseous torticollis, or at the very least, limited rotation and lateral flexion, and be a source of cervicogenic headache, of numerous potential aetiologies corresponding to its anatomic relationships.

The arthrosis that may form with the epitransverse process of C1 at the base of the bony strut may be a source of pain, and CT-guided corticosteroid injection of this articulation may be efficacious [6].

The vertebral artery (VA) and adjacent vertebral vein relationships of the PCP have received little attention in the literature, and are depicted in Figs. 1 and 2. A close relationship of the PCP to the vertebral artery was inferred in an assessment of the calvarium, where the PCP was reported to be 0.42 mm from the occipital condyle [2]. From imaging assessment, the VA is an immediate medial and posterosuperiorly directed relation of the PCP, and the principal structure of concern if surgical resection is considered. Though the vessel can be seen on soft tissue CT windows, as in this case, it can be even better demonstrated on CT angiography or using intraoperative ultrasound, if surgery is to be contemplated.

A three-dimensional reconstruction was modelled from CT volume data, utilising 3D Slicer software [4]. Bony tissue was segmented initially via seeded, Fast-GrowCut algorithmic construction (‘Grow from seeds’), followed by detailed manual segmentation at tissue margins. Beyond the region of interest, certain interfaces of ambiguous density were clarified for illustrative purposes (namely, the odontoid process was partitioned from surrounding ligamentous tissue, and vertebral bony tissue was delineated from regions of marked sclerosis at lateral facets C2-C5). A 3D mesh was manually constructed to follow close reading of the path of the VA medial to the PCP. The resulting model is presented, illustrating the intimacy between the course of the VA and the articulated PCP [Figs. 1 and 2].

Further, the PCP additionally has important anatomical relationship with the suboccipital nerve. At the posteromedial aspect of the PCP, the vertebral artery is rostrally related to the dorsal ramus of C1 (the suboccipital nerve), passing along the suboccipital groove towards the suboccipital triangle muscles, and providing sensory afferents for the capsule of the atlanto-occipital joint.

Though C1 is said to possess no dermatome, a study of 30 cadavers showed that almost 30% of the 14 specimens with a C1 dorsal root possessed a dorsal root ganglion, suggesting a significant sensory role [11]. In addition, the suboccipital groove is said to house the ampulloglomerular organ, involved in pressure regulation of the complex vertebral venous plexus arrangement of the region, disorders of which have been implicated in headache pathophysiology [12]. Suboccipital nerve injury may result from surgical procedures of the posterior neck at the C1 and C2 levels, including posterior approaches for cervical neck fusions [3]. Damage to the suboccipital nerve may play a role in cervicogenic headaches and occipital neuralgia and thus is a structure that must be considered if surgical resection is contemplated.

The jugular vein is an anterolateral relation of the PCP, and lies superficial to rectus capitis lateralis (RCL), the muscle sweeping toward its C1 transverse process insertion, with some potential for insertion of some RCL muscle fibres into the PCP itself.

The presence of a PCP needs consideration in the differential diagnosis for neck pain with limited lateral flexion, with conservative management, image-guided steroid injection and neurosurgical resection of the PCP being the escalating treatment options. The PCP may be seen on AP plain radiographs, but is best depicted on CT. More frequently, the detection of PCP is an incidental finding. The anatomical relationships of the PCP, particularly that of the VA, are worthy of careful attention, given their important symptomatic and neurosurgical implications.

## Conclusion

This case demonstrates the anatomical and clinical significance of the paracondylar process (PCP), with a particular focus on its relationship with the vertebral artery. This finding carries important clinical implications if surgical resection is to be contemplated.

## Supplementary Information

Below is the link to the electronic supplementary material.

Supplementary Material 1

## Data Availability

No datasets were generated or analysed during the current study.
